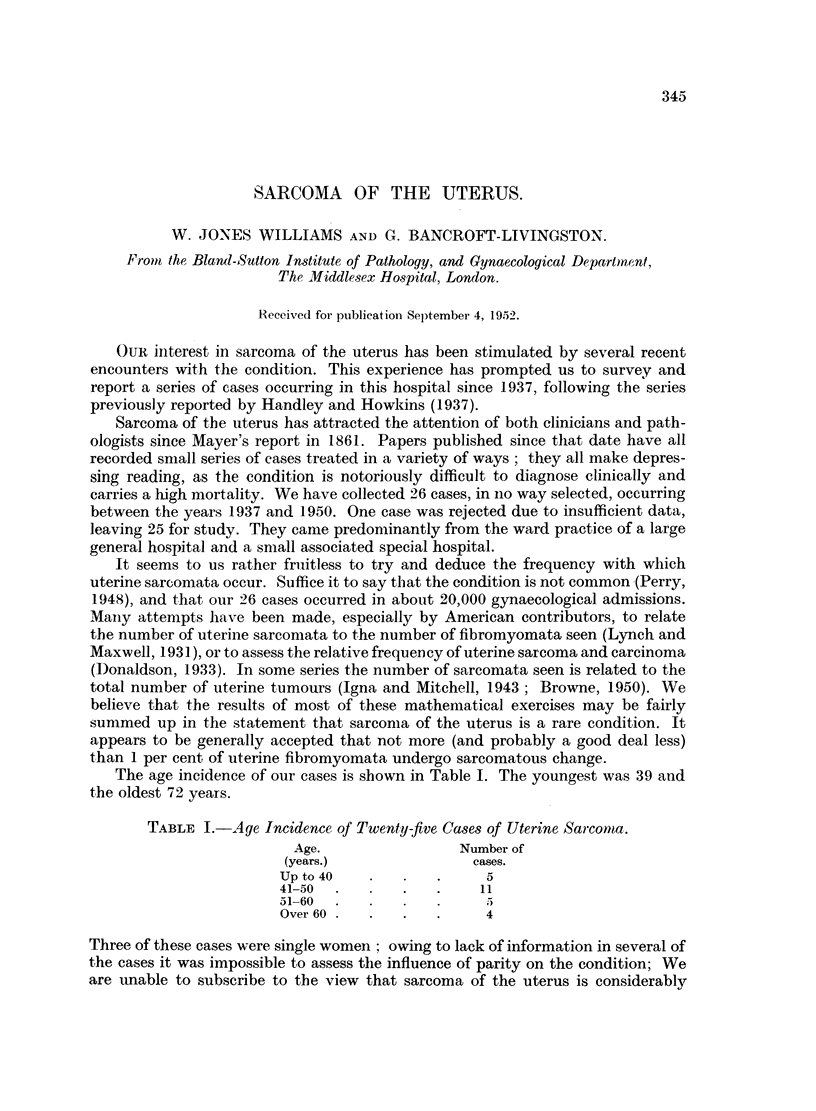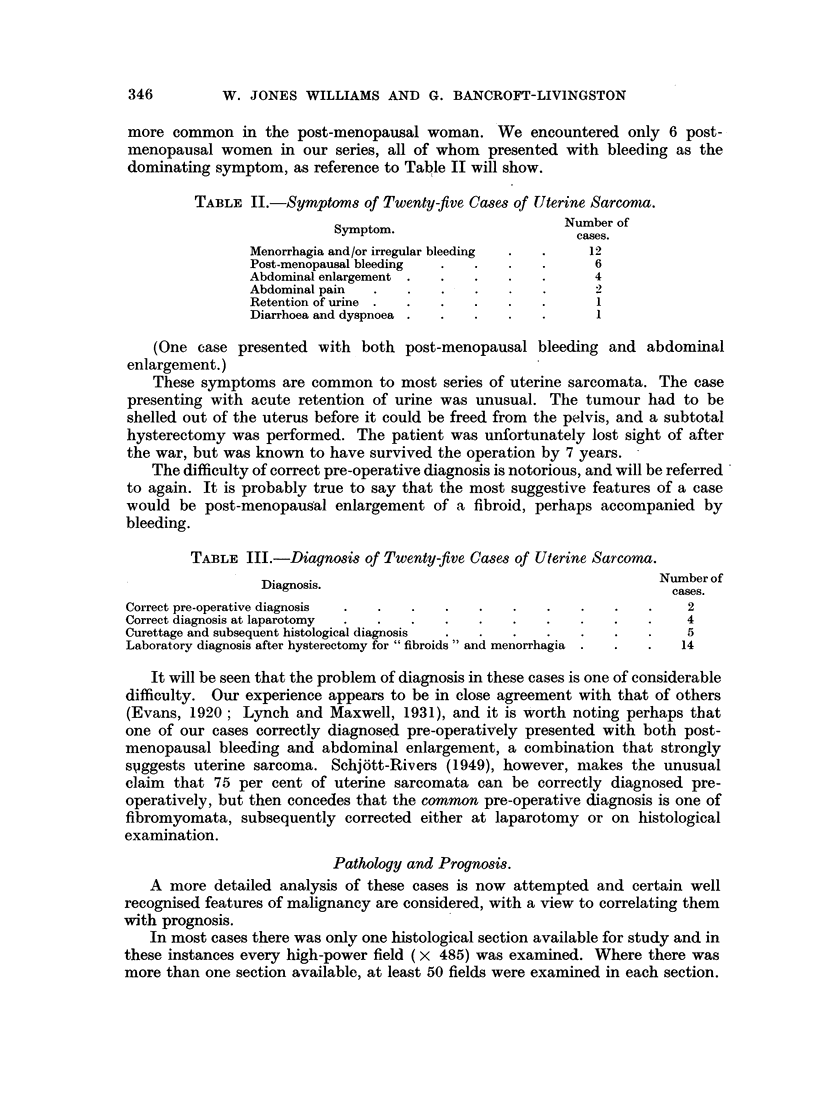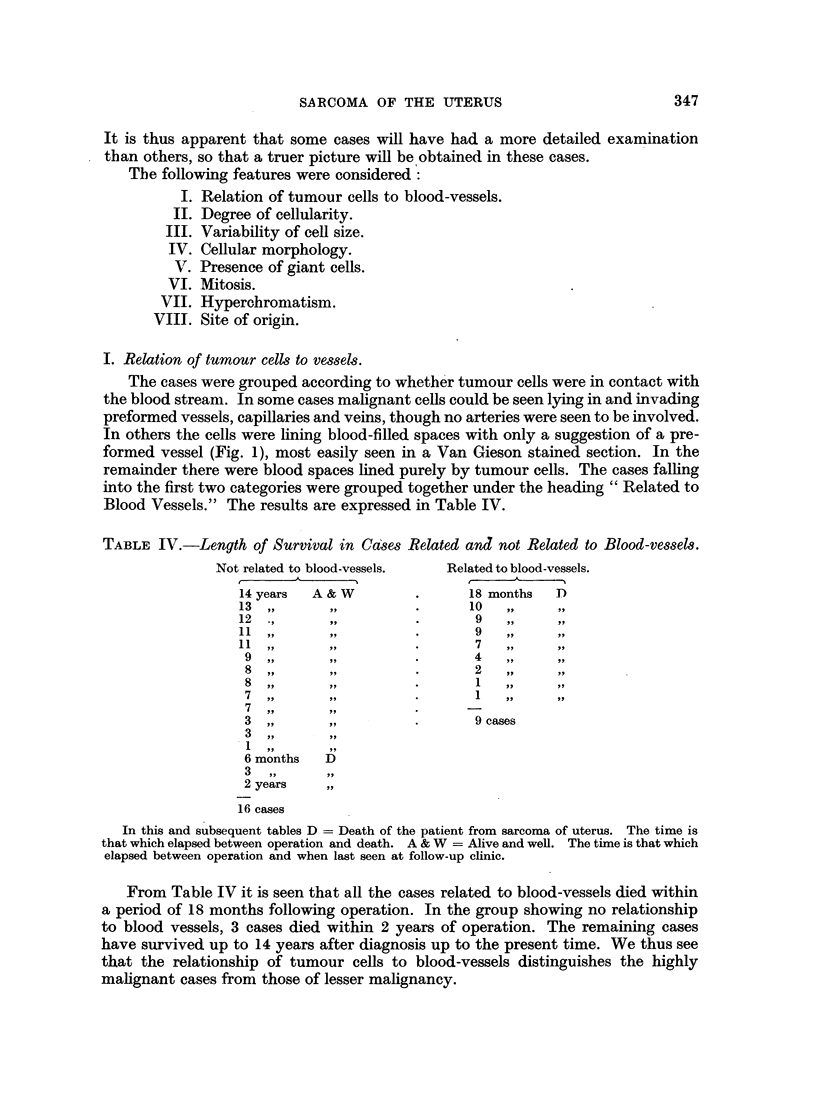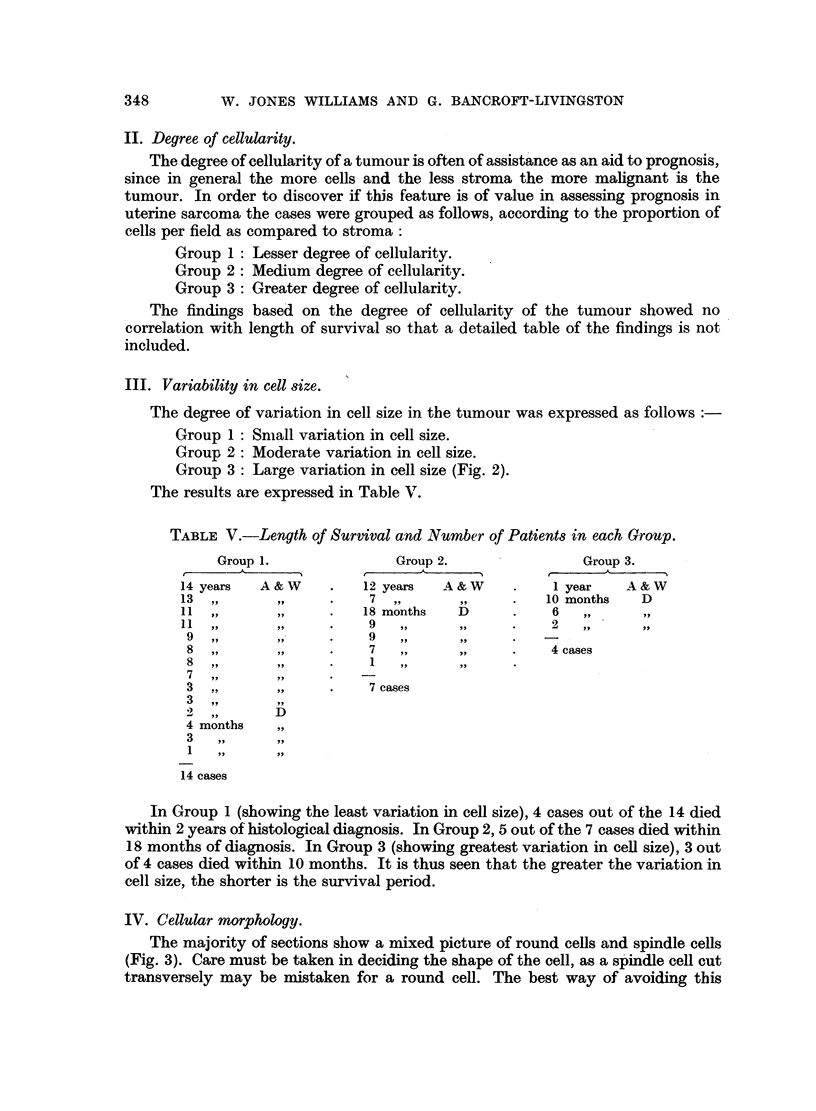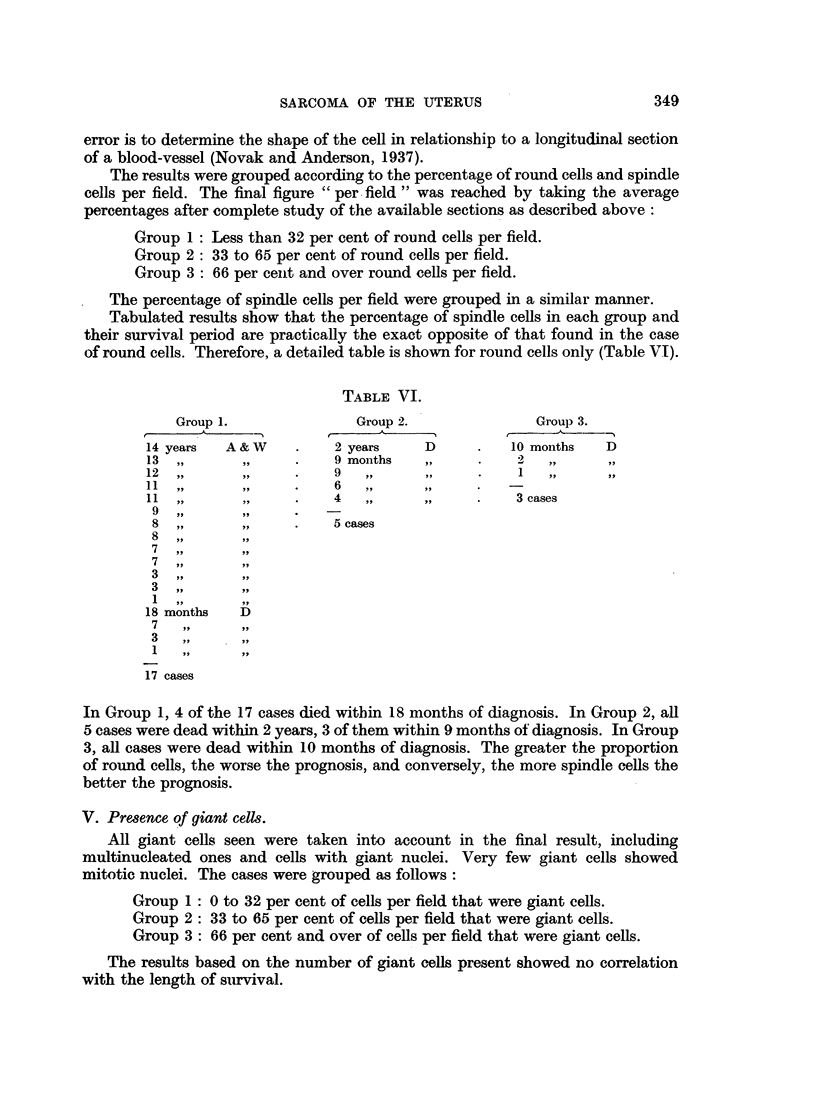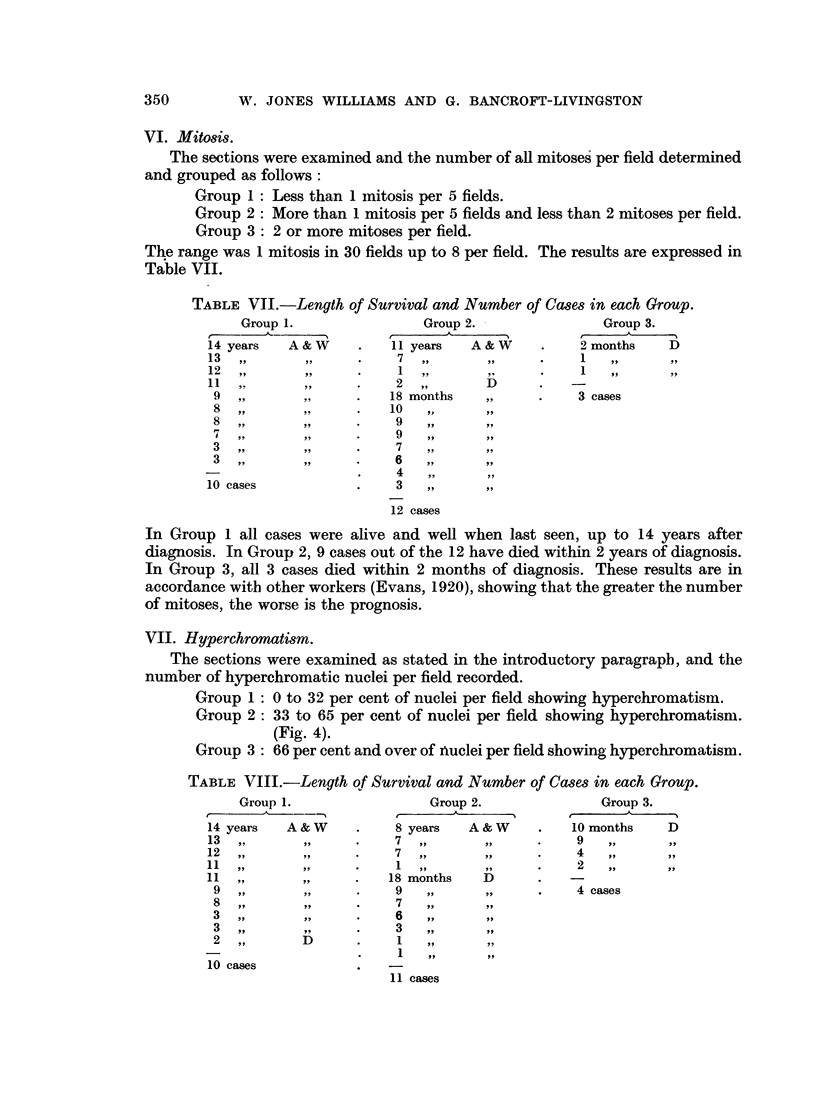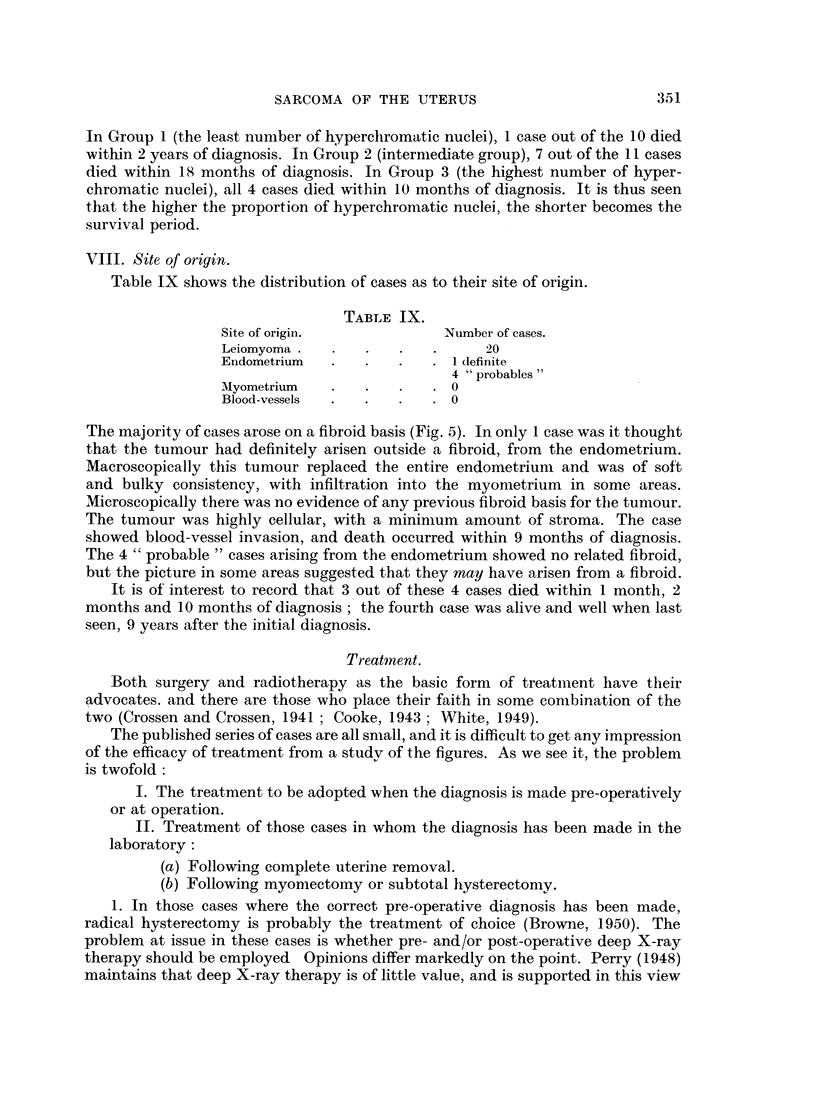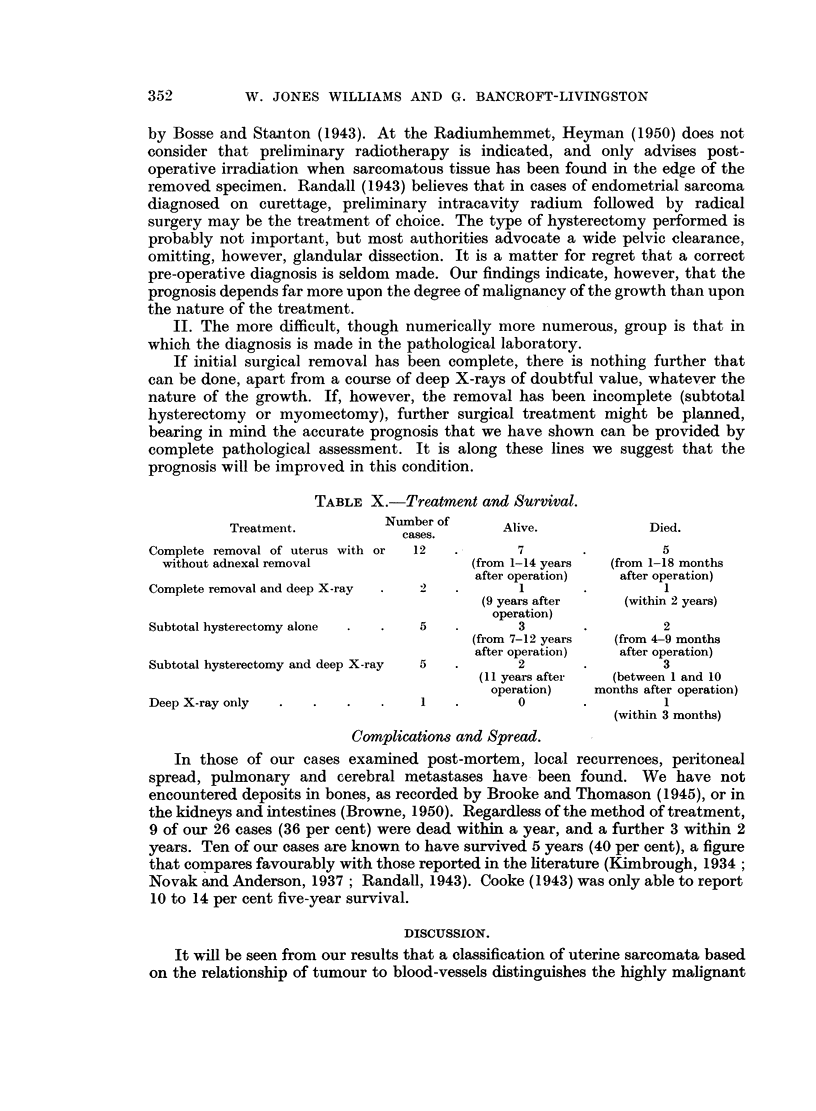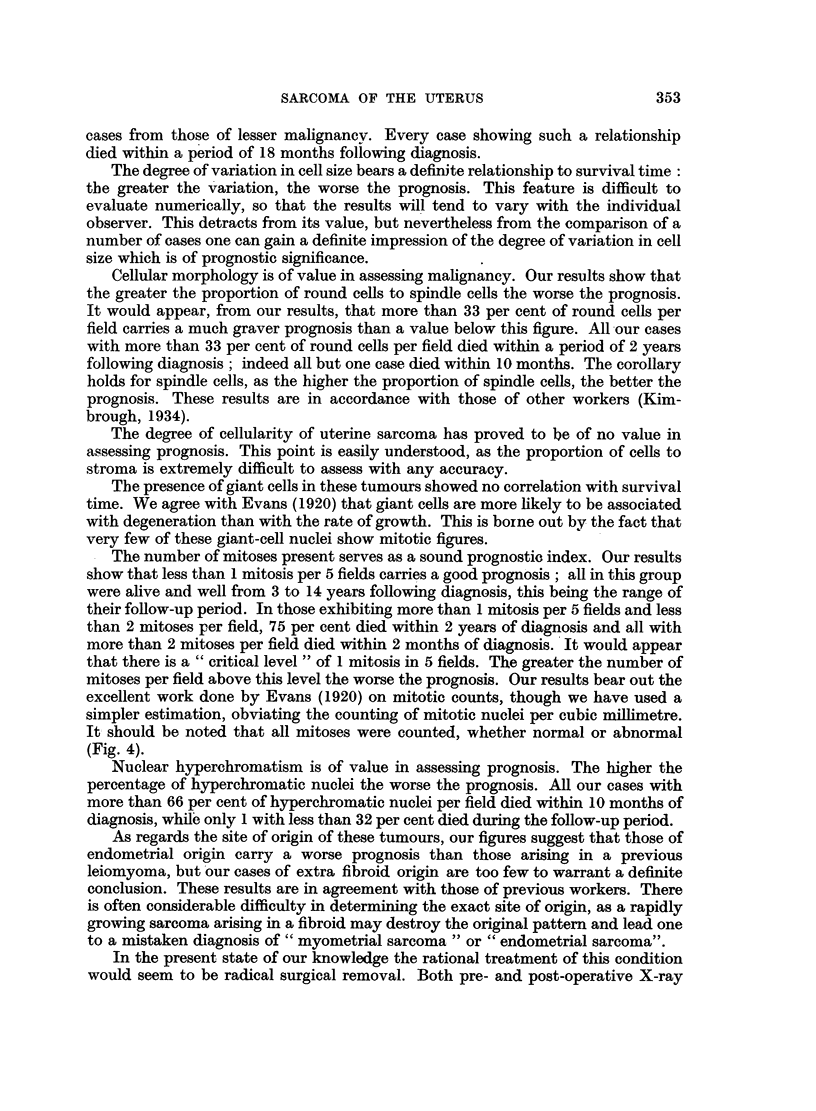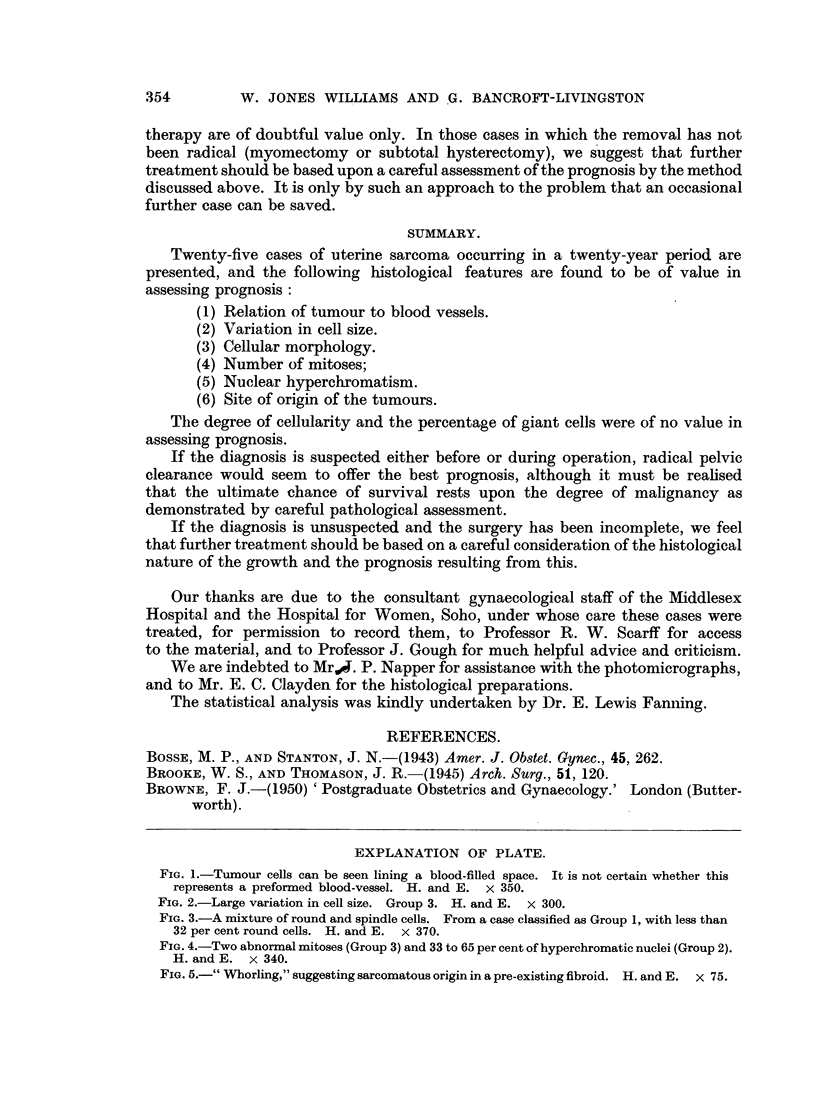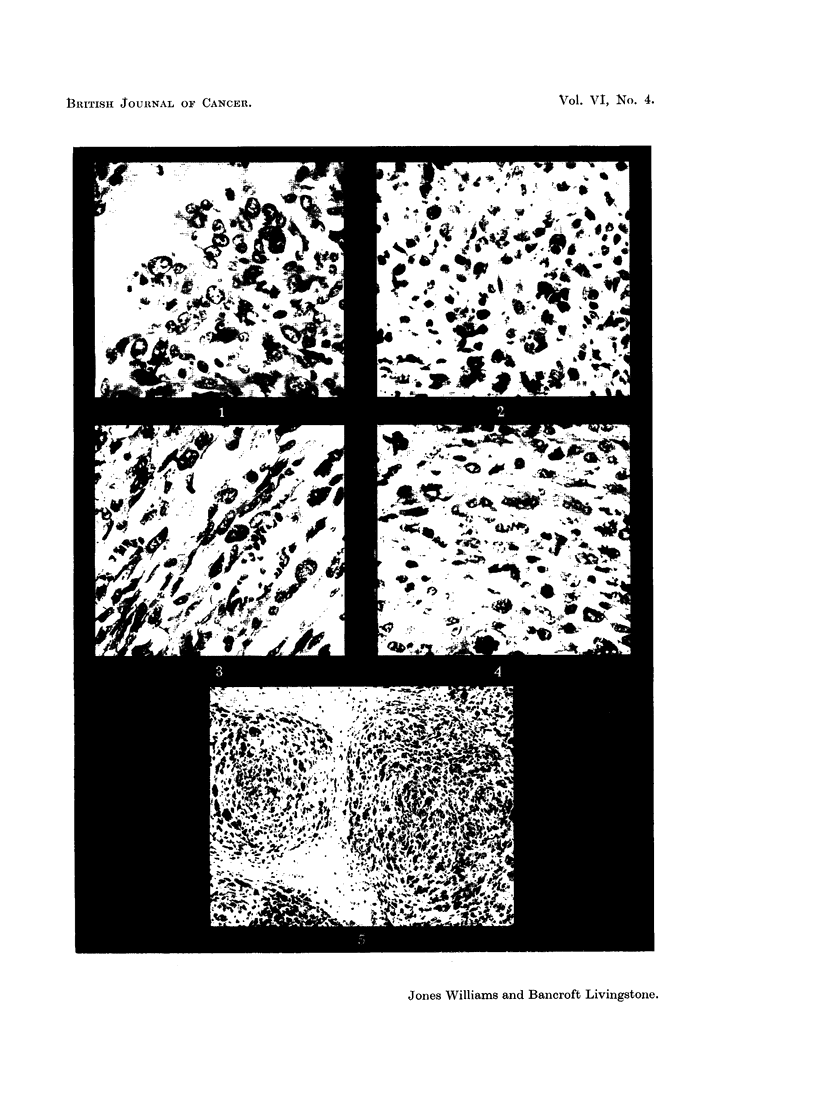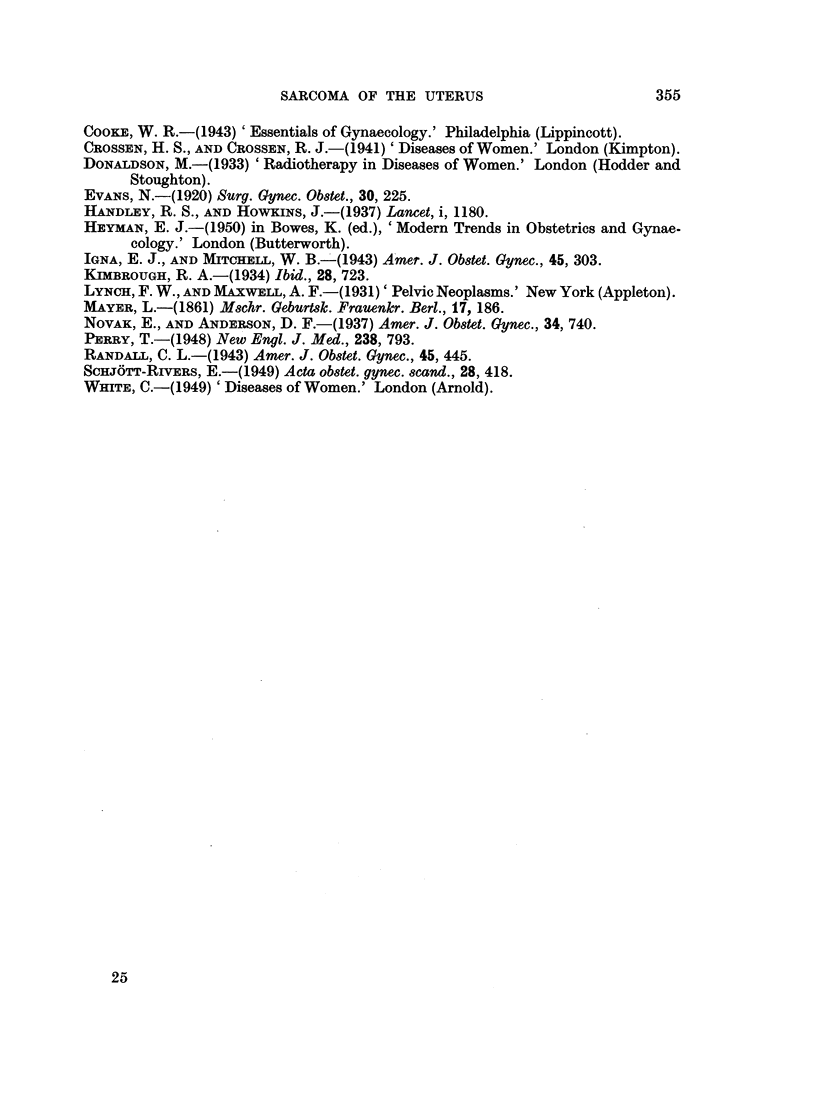# Sarcoma of the Uterus

**DOI:** 10.1038/bjc.1952.38

**Published:** 1952-12

**Authors:** W. Jones Williams, G. Bancroft-Livingston

## Abstract

**Images:**


### SARCOMA OF THE UTERUS.

W. JONES WILLIAMS AND G. BANCROFT-LIVINGSTON.

*From the Bland-Sutton Institute of Pathology, and Gynaecological Department, The Middlesex Hospital, London.*

Received for publication September 4, 1952.

OUR interest in sarcoma of the uterus has been stimulated by several recent encounters with the condition. This experience has prompted us to survey and report a series of cases occurring in this hospital since 1937, following the series previously reported by Handley and Howkins (1937).

Sarcoma of the uterus has attracted the attention of both clinicians and pathologists since Mayer's report in 1861. Papers published since that date have all recorded small series of cases treated in a variety of ways ; they all make depressing reading, as the condition is notoriously difficult to diagnose clinically and carries a high mortality. We have collected 26 cases, in no way selected, occurring between the years 1937 and 1950. One case was rejected due to insufficient data, leaving 25 for study. They came predominantly from the ward practice of a large general hospital and a small associated special hospital.

It seems to us rather fruitless to try and deduce the frequency with which uterine sarcomata occur. Suffice it to say that the condition is not common (Perry, 1948), and that our 26 cases occurred in about 20,000 gynaecological admissions. Many attempts have been made, especially by American contributors, to relate the number of uterine sarcomata to the number of fibromyomata seen (Lynch and Maxwell, 1931), or to assess the relative frequency of uterine sarcoma and carcinoma (Donaldson, 1933). In some series the number of sarcomata seen is related to the total number of uterine tumours (Igna and Mitchell, 1943 ; Browne, 1950). We believe that the results of most of these mathematical exercises may be fairly summed up in the statement that sarcoma of the uterus is a rare condition. It appears to be generally accepted that not more (and probably a good deal less) than 1 per cent of uterine fibromyomata undergo sarcomatous change.

The age incidence of our cases is shown in Table I. The youngest was 39 and the oldest 72 years.

TABLE I.—*Age Incidence of Twenty-five Cases of Uterine Sarcoma.*

| Age. (years.) | Number of cases. |
|---|---|
| Up to 40 | 5 |
| 41–50 | 11 |
| 51–60 | 5 |
| Over 60 | 4 |

Three of these cases were single women ; owing to lack of information in several of the cases it was impossible to assess the influence of parity on the condition; We are unable to subscribe to the view that sarcoma of the uterus is considerably more common in the post-menopausal woman. We encountered only 6 post-menopausal women in our series, all of whom presented with bleeding as the dominating symptom, as reference to Table II will show.

TABLE II.—*Symptoms of Twenty-five Cases of Uterine Sarcoma.*

| Symptom. | Number of cases. |
|---|---|
| Menorrhagia and/or irregular bleeding | 12 |
| Post-menopausal bleeding | 6 |
| Abdominal enlargement | 4 |
| Abdominal pain | 2 |
| Retention of urine | 1 |
| Diarrhoea and dyspnoea | 1 |

(One case presented with both post-menopausal bleeding and abdominal enlargement.)

These symptoms are common to most series of uterine sarcomata. The case presenting with acute retention of urine was unusual. The tumour had to be shelled out of the uterus before it could be freed from the pelvis, and a subtotal hysterectomy was performed. The patient was unfortunately lost sight of after the war, but was known to have survived the operation by 7 years.

The difficulty of correct pre-operative diagnosis is notorious, and will be referred to again. It is probably true to say that the most suggestive features of a case would be post-menopausal enlargement of a fibroid, perhaps accompanied by bleeding.

TABLE III.—*Diagnosis of Twenty-five Cases of Uterine Sarcoma.*

| Diagnosis. | Number of cases. |
|---|---|
| Correct pre-operative diagnosis | 2 |
| Correct diagnosis at laparotomy | 4 |
| Curettage and subsequent histological diagnosis | 5 |
| Laboratory diagnosis after hysterectomy for " fibroids " and menorrhagia | 14 |

It will be seen that the problem of diagnosis in these cases is one of considerable difficulty. Our experience appears to be in close agreement with that of others (Evans, 1920; Lynch and Maxwell, 1931), and it is worth noting perhaps that one of our cases correctly diagnosed pre-operatively presented with both post-menopausal bleeding and abdominal enlargement, a combination that strongly suggests uterine sarcoma. Schjött-Rivers (1949), however, makes the unusual claim that 75 per cent of uterine sarcomata can be correctly diagnosed pre-operatively, but then concedes that the *common* pre-operative diagnosis is one of fibromyomata, subsequently corrected either at laparotomy or on histological examination.

### *Pathology and Prognosis.*

A more detailed analysis of these cases is now attempted and certain well recognised features of malignancy are considered, with a view to correlating them with prognosis.

In most cases there was only one histological section available for study and in these instances every high-power field ($\times$ 485) was examined. Where there was more than one section available, at least 50 fields were examined in each section.

It is thus apparent that some cases will have had a more detailed examination than others, so that a truer picture will be obtained in these cases.

The following features were considered :

I. Relation of tumour cells to blood-vessels.
II. Degree of cellularity.
III. Variability of cell size.
IV. Cellular morphology.
V. Presence of giant cells.
VI. Mitosis.
VII. Hyperchromatism.
VIII. Site of origin.

I. *Relation of tumour cells to vessels.*

The cases were grouped according to whether tumour cells were in contact with the blood stream. In some cases malignant cells could be seen lying in and invading preformed vessels, capillaries and veins, though no arteries were seen to be involved. In others the cells were lining blood-filled spaces with only a suggestion of a preformed vessel (Fig. 1), most easily seen in a Van Gieson stained section. In the remainder there were blood spaces lined purely by tumour cells. The cases falling into the first two categories were grouped together under the heading " Related to Blood Vessels." The results are expressed in Table IV.

TABLE IV.—*Length of Survival in Cases Related and not Related to Blood-vessels.*

| Not related to blood-vessels. | | Related to blood-vessels. | |
|---|---|---|---|
| 14 years | A & W | 18 months | D |
| 13 ,, | ,, | 10 ,, | ,, |
| 12 ,, | ,, | 9 ,, | ,, |
| 11 ,, | ,, | 9 ,, | ,, |
| 11 ,, | ,, | 7 ,, | ,, |
| 9 ,, | ,, | 4 ,, | ,, |
| 8 ,, | ,, | 2 ,, | ,, |
| 8 ,, | ,, | 1 ,, | ,, |
| 7 ,, | ,, | 1 ,, | ,, |
| 7 ,, | ,, | — | |
| 3 ,, | ,, | 9 cases | |
| 3 ,, | ,, | | |
| 1 ,, | ,, | | |
| 6 months | D | | |
| 3 ,, | ,, | | |
| 2 years | ,, | | |
| — | | | |
| 16 cases | | | |

In this and subsequent tables D = Death of the patient from sarcoma of uterus. The time is that which elapsed between operation and death. A & W = Alive and well. The time is that which elapsed between operation and when last seen at follow-up clinic.

From Table IV it is seen that all the cases related to blood-vessels died within a period of 18 months following operation. In the group showing no relationship to blood vessels, 3 cases died within 2 years of operation. The remaining cases have survived up to 14 years after diagnosis up to the present time. We thus see that the relationship of tumour cells to blood-vessels distinguishes the highly malignant cases from those of lesser malignancy.

II. *Degree of cellularity.*

The degree of cellularity of a tumour is often of assistance as an aid to prognosis, since in general the more cells and the less stroma the more malignant is the tumour. In order to discover if this feature is of value in assessing prognosis in uterine sarcoma the cases were grouped as follows, according to the proportion of cells per field as compared to stroma :

Group 1 : Lesser degree of cellularity.
Group 2 : Medium degree of cellularity.
Group 3 : Greater degree of cellularity.

The findings based on the degree of cellularity of the tumour showed no correlation with length of survival so that a detailed table of the findings is not included.

III. *Variability in cell size.*

The degree of variation in cell size in the tumour was expressed as follows :—

Group 1 : Small variation in cell size.
Group 2 : Moderate variation in cell size.
Group 3 : Large variation in cell size (Fig. 2).

The results are expressed in Table V.

TABLE V.—*Length of Survival and Number of Patients in each Group.*

| Group 1. | | Group 2. | | Group 3. | |
|---|---|---|---|---|---|
| 14 years | A & W | 12 years | A & W | 1 year | A & W |
| 13 " | " | 7 " | " | 10 months | D |
| 11 " | " | 18 months | D | 6 " | " |
| 11 " | " | 9 " | " | 2 " | " |
| 9 " | " | 9 " | " | — | |
| 8 " | " | 7 " | " | 4 cases | |
| 8 " | " | 1 " | " | | |
| 7 " | " | — | | | |
| 3 " | " | 7 cases | | | |
| 3 " | " | | | | |
| 2 " | D | | | | |
| 4 months | " | | | | |
| 3 " | " | | | | |
| 1 " | " | | | | |
| — | | | | | |
| 14 cases | | | | | |

In Group 1 (showing the least variation in cell size), 4 cases out of the 14 died within 2 years of histological diagnosis. In Group 2, 5 out of the 7 cases died within 18 months of diagnosis. In Group 3 (showing greatest variation in cell size), 3 out of 4 cases died within 10 months. It is thus seen that the greater the variation in cell size, the shorter is the survival period.

IV. *Cellular morphology.*

The majority of sections show a mixed picture of round cells and spindle cells (Fig. 3). Care must be taken in deciding the shape of the cell, as a spindle cell cut transversely may be mistaken for a round cell. The best way of avoiding this error is to determine the shape of the cell in relationship to a longitudinal section of a blood-vessel (Novak and Anderson, 1937).

The results were grouped according to the percentage of round cells and spindle cells per field. The final figure " per field " was reached by taking the average percentages after complete study of the available sections as described above :

Group 1 : Less than 32 per cent of round cells per field.
Group 2 : 33 to 65 per cent of round cells per field.
Group 3 : 66 per cent and over round cells per field.

The percentage of spindle cells per field were grouped in a similar manner.

Tabulated results show that the percentage of spindle cells in each group and their survival period are practically the exact opposite of that found in the case of round cells. Therefore, a detailed table is shown for round cells only (Table VI).

TABLE VI.

| Group 1. | | Group 2. | | Group 3. | |
|---|---|---|---|---|---|
| 14 years | A & W | 2 years | D | 10 months | D |
| 13 " | " | 9 months | " | 2 " | " |
| 12 " | " | 9 " | " | 1 " | " |
| 11 " | " | 6 " | " | — | |
| 11 " | " | 4 " | " | 3 cases | |
| 9 " | " | — | | | |
| 8 " | " | 5 cases | | | |
| 8 " | " | | | | |
| 7 " | " | | | | |
| 7 " | " | | | | |
| 3 " | " | | | | |
| 3 " | " | | | | |
| 1 " | " | | | | |
| 18 months | D | | | | |
| 7 " | " | | | | |
| 3 " | " | | | | |
| 1 " | " | | | | |
| — | | | | | |
| 17 cases | | | | | |

In Group 1, 4 of the 17 cases died within 18 months of diagnosis. In Group 2, all 5 cases were dead within 2 years, 3 of them within 9 months of diagnosis. In Group 3, all cases were dead within 10 months of diagnosis. The greater the proportion of round cells, the worse the prognosis, and conversely, the more spindle cells the better the prognosis.

V. *Presence of giant cells.*

All giant cells seen were taken into account in the final result, including multinucleated ones and cells with giant nuclei. Very few giant cells showed mitotic nuclei. The cases were grouped as follows :

Group 1 : 0 to 32 per cent of cells per field that were giant cells.
Group 2 : 33 to 65 per cent of cells per field that were giant cells.
Group 3 : 66 per cent and over of cells per field that were giant cells.

The results based on the number of giant cells present showed no correlation with the length of survival.

VI. *Mitosis.*

The sections were examined and the number of all mitoses per field determined and grouped as follows :

Group 1 : Less than 1 mitosis per 5 fields.
Group 2 : More than 1 mitosis per 5 fields and less than 2 mitoses per field.
Group 3 : 2 or more mitoses per field.

The range was 1 mitosis in 30 fields up to 8 per field. The results are expressed in Table VII.

TABLE VII.—*Length of Survival and Number of Cases in each Group.*

| Group 1. | | Group 2. | | Group 3. | |
|---|---|---|---|---|---|
| 14 years | A & W | 11 years | A & W | 2 months | D |
| 13 ” | ” | 7 ” | ” | 1 ” | ” |
| 12 ” | ” | 1 ” | ” | 1 ” | ” |
| 11 ” | ” | 2 ” | D | — | |
| 9 ” | ” | 18 months | ” | 3 cases | |
| 8 ” | ” | 10 ” | ” | | |
| 8 ” | ” | 9 ” | ” | | |
| 7 ” | ” | 9 ” | ” | | |
| 3 ” | ” | 7 ” | ” | | |
| 3 ” | ” | 6 ” | ” | | |
| — | | 4 ” | ” | | |
| 10 cases | | 3 ” | ” | | |
| | | — | | | |
| | | 12 cases | | | |

In Group 1 all cases were alive and well when last seen, up to 14 years after diagnosis. In Group 2, 9 cases out of the 12 have died within 2 years of diagnosis. In Group 3, all 3 cases died within 2 months of diagnosis. These results are in accordance with other workers (Evans, 1920), showing that the greater the number of mitoses, the worse is the prognosis.

VII. *Hyperchromatism.*

The sections were examined as stated in the introductory paragraph, and the number of hyperchromatic nuclei per field recorded.

Group 1 : 0 to 32 per cent of nuclei per field showing hyperchromatism.
Group 2 : 33 to 65 per cent of nuclei per field showing hyperchromatism. (Fig. 4).
Group 3 : 66 per cent and over of nuclei per field showing hyperchromatism.

TABLE VIII.—*Length of Survival and Number of Cases in each Group.*

| Group 1. | | Group 2. | | Group 3. | |
|---|---|---|---|---|---|
| 14 years | A & W | 8 years | A & W | 10 months | D |
| 13 ” | ” | 7 ” | ” | 9 ” | ” |
| 12 ” | ” | 7 ” | ” | 4 ” | ” |
| 11 ” | ” | 1 ” | ” | 2 ” | ” |
| 11 ” | ” | 18 months | D | — | |
| 9 ” | ” | 9 ” | ” | 4 cases | |
| 8 ” | ” | 7 ” | ” | | |
| 3 ” | ” | 6 ” | ” | | |
| 3 ” | ” | 3 ” | ” | | |
| 2 ” | D | 1 ” | ” | | |
| — | | 1 ” | ” | | |
| 10 cases | | — | | | |
| | | 11 cases | | | |

In Group 1 (the least number of hyperchromatic nuclei), 1 case out of the 10 died within 2 years of diagnosis. In Group 2 (intermediate group), 7 out of the 11 cases died within 18 months of diagnosis. In Group 3 (the highest number of hyperchromatic nuclei), all 4 cases died within 10 months of diagnosis. It is thus seen that the higher the proportion of hyperchromatic nuclei, the shorter becomes the survival period.

VIII. *Site of origin.*

Table IX shows the distribution of cases as to their site of origin.

TABLE IX.

| Site of origin. | Number of cases. |
|---|---|
| Leiomyoma | 20 |
| Endometrium | 1 definite<br>4 " probables " |
| Myometrium | 0 |
| Blood-vessels | 0 |

The majority of cases arose on a fibroid basis (Fig. 5). In only 1 case was it thought that the tumour had definitely arisen outside a fibroid, from the endometrium. Macroscopically this tumour replaced the entire endometrium and was of soft and bulky consistency, with infiltration into the myometrium in some areas. Microscopically there was no evidence of any previous fibroid basis for the tumour. The tumour was highly cellular, with a minimum amount of stroma. The case showed blood-vessel invasion, and death occurred within 9 months of diagnosis. The 4 " probable " cases arising from the endometrium showed no related fibroid, but the picture in some areas suggested that they *may* have arisen from a fibroid.

It is of interest to record that 3 out of these 4 cases died within 1 month, 2 months and 10 months of diagnosis ; the fourth case was alive and well when last seen, 9 years after the initial diagnosis.

*Treatment.*

Both surgery and radiotherapy as the basic form of treatment have their advocates. and there are those who place their faith in some combination of the two (Crossen and Crossen, 1941 ; Cooke, 1943 ; White, 1949).

The published series of cases are all small, and it is difficult to get any impression of the efficacy of treatment from a study of the figures. As we see it, the problem is twofold :

I. The treatment to be adopted when the diagnosis is made pre-operatively or at operation.

II. Treatment of those cases in whom the diagnosis has been made in the laboratory :

(*a*) Following complete uterine removal.

(*b*) Following myomectomy or subtotal hysterectomy.

1. In those cases where the correct pre-operative diagnosis has been made, radical hysterectomy is probably the treatment of choice (Browne, 1950). The problem at issue in these cases is whether pre- and/or post-operative deep X-ray therapy should be employed Opinions differ markedly on the point. Perry (1948) maintains that deep X-ray therapy is of little value, and is supported in this view by Bosse and Stanton (1943). At the Radiumhemmet, Heyman (1950) does not consider that preliminary radiotherapy is indicated, and only advises post-operative irradiation when sarcomatous tissue has been found in the edge of the removed specimen. Randall (1943) believes that in cases of endometrial sarcoma diagnosed on curettage, preliminary intracavity radium followed by radical surgery may be the treatment of choice. The type of hysterectomy performed is probably not important, but most authorities advocate a wide pelvic clearance, omitting, however, glandular dissection. It is a matter for regret that a correct pre-operative diagnosis is seldom made. Our findings indicate, however, that the prognosis depends far more upon the degree of malignancy of the growth than upon the nature of the treatment.

II. The more difficult, though numerically more numerous, group is that in which the diagnosis is made in the pathological laboratory.

If initial surgical removal has been complete, there is nothing further that can be done, apart from a course of deep X-rays of doubtful value, whatever the nature of the growth. If, however, the removal has been incomplete (subtotal hysterectomy or myomectomy), further surgical treatment might be planned, bearing in mind the accurate prognosis that we have shown can be provided by complete pathological assessment. It is along these lines we suggest that the prognosis will be improved in this condition.

TABLE X.—*Treatment and Survival.*

| Treatment. | Number of cases. | Alive. | Died. |
|---|---|---|---|
| Complete removal of uterus with or without adnexal removal | 12 | 7 (from 1–14 years after operation) | 5 (from 1–18 months after operation) |
| Complete removal and deep X-ray | 2 | 1 (9 years after operation) | 1 (within 2 years) |
| Subtotal hysterectomy alone | 5 | 3 (from 7–12 years after operation) | 2 (from 4–9 months after operation) |
| Subtotal hysterectomy and deep X-ray | 5 | 2 (11 years after operation) | 3 (between 1 and 10 months after operation) |
| Deep X-ray only | 1 | 0 | 1 (within 3 months) |

*Complications and Spread.*

In those of our cases examined post-mortem, local recurrences, peritoneal spread, pulmonary and cerebral metastases have been found. We have not encountered deposits in bones, as recorded by Brooke and Thomason (1945), or in the kidneys and intestines (Browne, 1950). Regardless of the method of treatment, 9 of our 26 cases (36 per cent) were dead within a year, and a further 3 within 2 years. Ten of our cases are known to have survived 5 years (40 per cent), a figure that compares favourably with those reported in the literature (Kimbrough, 1934 ; Novak and Anderson, 1937 ; Randall, 1943). Cooke (1943) was only able to report 10 to 14 per cent five-year survival.

### DISCUSSION.

It will be seen from our results that a classification of uterine sarcomata based on the relationship of tumour to blood-vessels distinguishes the highly malignant cases from those of lesser malignancy. Every case showing such a relationship died within a period of 18 months following diagnosis.

The degree of variation in cell size bears a definite relationship to survival time : the greater the variation, the worse the prognosis. This feature is difficult to evaluate numerically, so that the results will tend to vary with the individual observer. This detracts from its value, but nevertheless from the comparison of a number of cases one can gain a definite impression of the degree of variation in cell size which is of prognostic significance.

Cellular morphology is of value in assessing malignancy. Our results show that the greater the proportion of round cells to spindle cells the worse the prognosis. It would appear, from our results, that more than 33 per cent of round cells per field carries a much graver prognosis than a value below this figure. All our cases with more than 33 per cent of round cells per field died within a period of 2 years following diagnosis ; indeed all but one case died within 10 months. The corollary holds for spindle cells, as the higher the proportion of spindle cells, the better the prognosis. These results are in accordance with those of other workers (Kimbrough, 1934).

The degree of cellularity of uterine sarcoma has proved to be of no value in assessing prognosis. This point is easily understood, as the proportion of cells to stroma is extremely difficult to assess with any accuracy.

The presence of giant cells in these tumours showed no correlation with survival time. We agree with Evans (1920) that giant cells are more likely to be associated with degeneration than with the rate of growth. This is borne out by the fact that very few of these giant-cell nuclei show mitotic figures.

The number of mitoses present serves as a sound prognostic index. Our results show that less than 1 mitosis per 5 fields carries a good prognosis ; all in this group were alive and well from 3 to 14 years following diagnosis, this being the range of their follow-up period. In those exhibiting more than 1 mitosis per 5 fields and less than 2 mitoses per field, 75 per cent died within 2 years of diagnosis and all with more than 2 mitoses per field died within 2 months of diagnosis. It would appear that there is a " critical level " of 1 mitosis in 5 fields. The greater the number of mitoses per field above this level the worse the prognosis. Our results bear out the excellent work done by Evans (1920) on mitotic counts, though we have used a simpler estimation, obviating the counting of mitotic nuclei per cubic millimetre. It should be noted that all mitoses were counted, whether normal or abnormal (Fig. 4).

Nuclear hyperchromatism is of value in assessing prognosis. The higher the percentage of hyperchromatic nuclei the worse the prognosis. All our cases with more than 66 per cent of hyperchromatic nuclei per field died within 10 months of diagnosis, while only 1 with less than 32 per cent died during the follow-up period.

As regards the site of origin of these tumours, our figures suggest that those of endometrial origin carry a worse prognosis than those arising in a previous leiomyoma, but our cases of extra fibroid origin are too few to warrant a definite conclusion. These results are in agreement with those of previous workers. There is often considerable difficulty in determining the exact site of origin, as a rapidly growing sarcoma arising in a fibroid may destroy the original pattern and lead one to a mistaken diagnosis of " myometrial sarcoma " or " endometrial sarcoma".

In the present state of our knowledge the rational treatment of this condition would seem to be radical surgical removal. Both pre- and post-operative X-ray therapy are of doubtful value only. In those cases in which the removal has not been radical (myomectomy or subtotal hysterectomy), we suggest that further treatment should be based upon a careful assessment of the prognosis by the method discussed above. It is only by such an approach to the problem that an occasional further case can be saved.

### SUMMARY.

Twenty-five cases of uterine sarcoma occurring in a twenty-year period are presented, and the following histological features are found to be of value in assessing prognosis :

(1) Relation of tumour to blood vessels.
(2) Variation in cell size.
(3) Cellular morphology.
(4) Number of mitoses;
(5) Nuclear hyperchromatism.
(6) Site of origin of the tumours.

The degree of cellularity and the percentage of giant cells were of no value in assessing prognosis.

If the diagnosis is suspected either before or during operation, radical pelvic clearance would seem to offer the best prognosis, although it must be realised that the ultimate chance of survival rests upon the degree of malignancy as demonstrated by careful pathological assessment.

If the diagnosis is unsuspected and the surgery has been incomplete, we feel that further treatment should be based on a careful consideration of the histological nature of the growth and the prognosis resulting from this.

Our thanks are due to the consultant gynaecological staff of the Middlesex Hospital and the Hospital for Women, Soho, under whose care these cases were treated, for permission to record them, to Professor R. W. Scarff for access to the material, and to Professor J. Gough for much helpful advice and criticism.

We are indebted to Mr. J. P. Napper for assistance with the photomicrographs, and to Mr. E. C. Clayden for the histological preparations.

The statistical analysis was kindly undertaken by Dr. E. Lewis Fanning.

### REFERENCES.

BOSSE, M. P., AND STANTON, J. N.—(1943) *Amer. J. Obstet. Gynec.*, **45**, 262.
BROOKE, W. S., AND THOMASON, J. R.—(1945) *Arch. Surg.*, **51**, 120.
BROWNE, F. J.—(1950) 'Postgraduate Obstetrics and Gynaecology.' London (Butterworth).

---

### EXPLANATION OF PLATE.

FIG. 1.—Tumour cells can be seen lining a blood-filled space. It is not certain whether this represents a preformed blood-vessel. H. and E. × 350.

FIG. 2.—Large variation in cell size. Group 3. H. and E. × 300.

FIG. 3.—A mixture of round and spindle cells. From a case classified as Group 1, with less than 32 per cent round cells. H. and E. × 370.

FIG. 4.—Two abnormal mitoses (Group 3) and 33 to 65 per cent of hyperchromatic nuclei (Group 2). H. and E. × 340.

FIG. 5.—" Whorling," suggesting sarcomatous origin in a pre-existing fibroid. H. and E. × 75.

Jones Williams and Bancroft Livingstone.

COOKE, W. R.—(1943) 'Essentials of Gynaecology.' Philadelphia (Lippincott).
CROSSEN, H. S., AND CROSSEN, R. J.—(1941) 'Diseases of Women.' London (Kimpton).
DONALDSON, M.—(1933) 'Radiotherapy in Diseases of Women.' London (Hodder and Stoughton).
EVANS, N.—(1920) *Surg. Gynec. Obstet.*, **30**, 225.
HANDLEY, R. S., AND HOWKINS, J.—(1937) *Lancet*, i, 1180.
HEYMAN, E. J.—(1950) in Bowes, K. (ed.), 'Modern Trends in Obstetrics and Gynaecology.' London (Butterworth).
IGNA, E. J., AND MITCHELL, W. B.—(1943) *Amer. J. Obstet. Gynec.*, **45**, 303.
KIMBROUGH, R. A.—(1934) *Ibid.*, **28**, 723.
LYNCH, F. W., AND MAXWELL, A. F.—(1931) 'Pelvic Neoplasms.' New York (Appleton).
MAYER, L.—(1861) *Mschr. Geburtsk. Frauenkr. Berl.*, **17**, 186.
NOVAK, E., AND ANDERSON, D. F.—(1937) *Amer. J. Obstet. Gynec.*, **34**, 740.
PERRY, T.—(1948) *New Engl. J. Med.*, **238**, 793.
RANDALL, C. L.—(1943) *Amer. J. Obstet. Gynec.*, **45**, 445.
SCHJÖTT-RIVERS, E.—(1949) *Acta obstet. gynec. scand.*, **28**, 418.
WHITE, C.—(1949) 'Diseases of Women.' London (Arnold).